# Ten-year trend in stroke incidence and its subtypes in Isfahan, Iran during 2003-2013

**Published:** 2017-10-07

**Authors:** Ahmad Bahonar, Alireza Khosravi, Fariborz Khorvash, Mohammadreza Maracy, Shahram Oveisgharan, Noushin Mohammadifard, Mohammad Saadatnia, Fatemeh Nouri, Nizal Sarrafzadegan

**Affiliations:** 1 Isfahan Neurosciences Research Center, Alzahra Hospital, Isfahan University of Medical Sciences, Isfahan, Iran; 2 Interventional Cardiology Research Center, Cardiovascular Research Institute, Isfahan University of Medical Sciences, Isfahan, Iran; 3 Department of Epidemiology and Biostatistics, School of Health, Isfahan University of Medical Sciences, Isfahan, Iran; 4 Shariati Hospital, Tehran University of Medical Sciences, Tehran, Iran; 5 Rush Alzheimer's Disease Center, Rush University of Medical Sciences, Chicago, IL, USA; 6 Hypertension Research Center, Cardiovascular Research Institute, Isfahan University of Medical Sciences, Isfahan, Iran; 7 Isfahan Cardiovascular Research Center, Cardiovascular Research Institute, Isfahan University of Medical Sciences, Isfahan, Iran

**Keywords:** Risk Factors, Incidence, Mortality, Stroke, Trend

## Abstract

**Background:** As there was no evidence of long-term studies on stroke trend, stroke subtypes and its relationships to stroke risk factors and demographic characteristics in Iran, we aimed to evaluate the 10-year trend of stroke incidence and stroke subtypes in Isfahan, Iran.

**Methods:** In a hospital-based retrospective study, 24186 cases with the first-ever stroke were analyzed. We assessed the incidence trend of annual stroke and its subtypes [ischemic stroke (IS) subarachnoid hemorrhage (SAH), and intracranial hemorrhage (ICH)] during the years 2003 to 2013 by sex, and studied the association of demographic and major stroke risk factors with incidence and mortality rate of stroke.

**Results:** The mean age was 69.46 ± 14.87 years, and 49.29% of patients were women. IS was the most frequent type among all the types of strokes (76.18%). Stroke and its subtypes had decreasing incidence trend during the study period, except for SAH that increased. In addition, stroke and its subtypes had decreasing mortality trend during the study period, except for SAH that did not change anymore. Stroke mortality and incidence rates were lower in urban inhabitants compared to residents of rural areas [odds ratio (OR) = 0.763, P < 0.001].

**Conclusion:** Despite the relatively high incidence of stroke over the study period, the incidence rate of stroke, especially ICH subtype, had a decreasing trend over the last decade in Isfahan. However, given the current young population in Iran, we can expect that the incidence of stroke would have an escalating trend in future.

## Introduction

Stroke is the second most common cause of mortality and disability worldwide.^1^ With increasing older population in the world, the increased incidence of stroke is predictable.^2^ On the other hand, low and middle-income countries had 100% increase in stroke deaths during the past four decades.^3^ Furthermore, evidence from the epidemiological trend in stroke and its subtypes worldwide indicates differences in the incidence and mortality rate of stroke by sex,^4^ urban/rural area of residence,^5^ and its variability trends over time.^6^

According to 2011 national census, over 60-year old population of Iran has increased from 7.22% in 2006 to 8.20%.^7^ Apart from demographic change, in the last decade, Iran has been facing profound changes in lifestyle that all of these factors together may be effective in increasing the incidence of stroke and mortality over time. Data from a recent study indicated high stroke incidence and mortality rates in Iran, demonstrating that the crude annual incidence rate of stroke in Iran were 144 and 133 per 100000 in men and in women, respectively.^8^

To our knowledge, there is little information about incidence and trend of stroke in Iranian population taken an explicit systematic approach for long-period studies. Bearing it in mind, we studied stroke incidence, mortality and major types by age in both sexes in Isfahan, Iran, and assessed whether the area of residence (i.e. rural vs. urban) contributed to the variation in stroke incidence and mortality among these populations. Analysis of existing data was necessary to identify a current trend in stroke epidemiology and its subtypes in this part of the world. This could be a basis for later evidence-based prevention programs.

## Materials and Methods

This was a hospital-based retrospective cross-sectional study conducted in the Neuroscience Research Center, Isfahan, in collaboration with the Cardiovascular Research Institute, Isfahan University of Medical Sciences. The patients with their first stroke during 2003 until the end of 2013, who were admitted to eight hospitals with departments of neurology, neurosurgery, and intensive care unit (ICU) located in Isfahan, were enrolled in the study. Among these patients, only first ischemic stroke (IS), first hemorrhagic stroke, or first nonspecific stroke for a participant was retained.

Data were collected following the World Health Organization (WHO) stepwise approach to stroke surveillance (STEPS-stroke) protocol.^9^ In the first step, we collected information on patients with stroke admitted to health facilities. Strokes, according to WHO definition, were diagnosed^10^ and classified into three subtypes, IS, intracranial hemorrhage (ICH), and subarachnoid hemorrhage (SAH) based on neuroimaging reports.^11^

In this study, three nurses who were educated before the study abstracted medical records. A statistician carried out the data entry. This study was reviewed and approved by the Human Ethics Committee, Neuroscience Research Center, Isfahan University of Medical Sciences.

First, the groups of women and men were compared in different aspects using Student's independent t- and chi-square tests. Quantitative and qualitative data were shown as the mean ± standard deviation (SD) and number (percentage), respectively. The incidence rate of stroke was directly standardized to the 5-year age distribution of Segi’s world population.^12^ Segi's world population was devised in the late 1950s by a cancer epidemiologist, Mitsuo Segi, based on the sum total of men and women populations of 46 countries in the 1950 publications of the WHO.^13^ The 95% confidence interval (CI) was calculated for stroke incidence rates. The incidence trend of stroke and its subtypes from 2003 to 2013 were calculated using linear regression. We also used logistic regression models to evaluate the association of sex, age, living area, and stroke subtypes and risk factors for stroke incidence rate and mortality. For all the tests, P less than 0.050 was considered statistically signiﬁcant. The term, incidence rate, was restricted to only the first stroke. Mortality rate was the number of fatal events that occur within 28 days per a population of 100000 people. All the data were analyzed using SPSS software (version 20, IBM Corporation, Armonk, NY, USA).

## Results

During this study from 2003 to 2013, in total, 24186 cases of stroke, including 11922 women (49.29%) and 12264 men (50.71%) were registered in Isfahan from eight hospitals. Patients' demographic characteristics, stroke type, and risk factors are shown in table 1.

It can be seen that the mean age was 69.46 ± 14.87 years and women were significantly older than men (69.99 ± 16.22 vs. 68.94 ± 13.39 years, respectively, P < 0.001). The highest percentage of patients was in the age group of above 65 years (66.53%). The frequency of patients aged more than 65 years was significantly higher among women than men [8103 (50.36%) vs. 7987 (49.64%), respectively, P < 0.001)]. 20675 (85.48%) and 3370 patients (13.93%) were residents of urban and rural areas, respectively. 

**Table 1:** Characteristics of men and women patients with stroke in Isfahan, Iran, between 2003 and 2013

**Characteristic**		**Total (n = 24186)**		**Women (n = 11922)**	**Men (n = 12264)**	**P** *
Age (year) (mean ± SD)			69.46 ± 14.87	69.99 ± 16.22	68.94 ± 13.39	< 0.001
Age group [n (%)]	< 45		1406 (5.81)	710 (50.50)	696 (49.50)	0.780
	45-65		6690 (27.66)	3109 (46.47)	3581 (53.53)	< 0.001
	> 65		16090 (66.53)	8103 (50.36)	7987 (49.64)	< 0.001
Living area [n (%)]	Urban		20675 (85.48)	10233 (49.49)	10442 (50.51)	0.670
	Rural		3370 (13.93)	1630 (48.37)	1740 (51.63)	0.310
	Undetermined		141(0.58)	-	-	-
Stroke type [n (%)]	IS		18425 (76.18)	9047 (49.10)	9378 (50.90)	0.690
	ICH		4334 (17.92)	2143 (49.45)	2191 (50.55)	0.850
	SAH		571 (2.36)	307 (53.77)	264 (46.23)	0.034
	Undetermined		856 (3.54)	-	-	-
Stroke risk factor [n (%)]	TIA		6160 (16.28)	2900 (47.08)	3260 (52.92)	< 0.001
	Diabetes		7417 (19.60)	3964 (53.44)	3453 (46.56)	< 0.001
	Elevated blood pressure		15890 (41.99)	8846 (55.67)	7044 (44.33)	< 0.001
	Hearth attack		8371 (22.12)	4289 (51.24)	4082 (48.78)	< 0.001

SD: Standard deviation; IS: Ischemic stroke; ICH: Intracranial hemorrhage; SAH: Subarachnoid hemorrhage; TIA: Transient ischemic attack

* Student's independent t- and chi-square tests (women vs. men)

There were no statistically signiﬁcant sex differences between stroke frequency in patients living in urban or rural areas (P = 0.670 and P = 0.310, respectively). IS was the most frequent subtype among all types of stroke, which the frequency of 18425 (76.18%). Moreover, SAH was statistically higher in women than in men [307 (53.77%) vs. 264 (46.23%), P = 0.030]. We did not find any statistically signiﬁcant difference in the number of other stroke subtypes between sexes. All stroke risk factors examined in this study were observed more frequently in women than in men (P < 0.001 for all), except transient ischemic attack (TIA).

Table 2 shows crude incidence rate (CIR) and age-adjusted incidence rate (AIR) (per 100000) of stroke types in Isfahan during 2003-2013. The occurrence of IS subtypes was highest compared with other subtypes. The age-adjusted occurrence of ICH (β = -3.29, P = 0.001) and IS (β = -5.63, P = 0.007) showed significantly decreasing trend during the study period. However, no significant change was observed in SAH subtype (β = 0.08, P = 0.450). 

**Table 2:** Age-adjusted incidence rate (AIR) (per 100000) of stroke types in Isfahan, Iran, during 2003-2013

**Year**	**Type of stroke**					
	**SAH [OR (95% CI)]**		**ICH [OR (95% CI)]**		**IS [OR (95% CI)]**	
	**CIR**	**AIR**	**CIR**	**AIR**	**CIR**	**AIR**
2003	3.7 (1.3-7.3)	4.9 (2.7-9.0)	37.3 (25.7-48.9)	53.8 (40.1-67.5)	108.6 (88.8-128.4)	157.0 (133.6-180.4)
2004	2.5 (0.5-5.5)	3.2 (0.2-6.6)	35.9 (24.5-47.3)	51.1 (37.7-64.5)	106.2 (86.6-125.8)	150.5 (127.5-173.5)
2005	3.0 (0.3-6.3)	3.7 (0.1-7.3)	27.3 (17.4-37.2)	38.4 (26.8-50.0)	118.6 (97.9-139.3)	165.3 (141.2-189.4)
2006	2.1 (0.7-4.9)	2.6 (0.4-5.8)	25.4 (15.8-35.0)	34.7 (23.6-45.8)	108.7 (88.9-128.5)	149.5 (126.5-172.5)
2007	2.0 (0.7-4.7)	2.5 (0.5-5.5)	20.5 (11.9-29.1)	27.7 (17.8-37.6)	86.2 (68.5-103.9)	115.2 (95.0-135.4)
2008	1.9 (0.7-4.5)	2.2 (0.6-5.0)	17.0 (9.2-24.8)	22.1 (13.2-31.0)	84.5 (67.0-102)	111.0 (91.1-130.9)
2009	2.5 (0.5-5.5)	2.8 (0.4-6.0)	13.6 (6.6-20.6)	17.4 (9.5-25.3)	69.1 (53.3-84.9)	89.6 (71.7-107.5)
2010	2.4 (0.6-5.4)	2.9 (0.3-6.1)	20.1 (11.6-28.6)	25.1 (15.6-34.6)	103.8 (84.4-123.2)	132.4 (110.7-154.1)
2011	4.1 (0.2-8.0)	4.8 (0.6-9.0)	18.6 (10.4-26.8)	23.0 (13.9-32.1)	90.5 (72.4-108.6)	112.9 (92.8-133.0)
2012	3.7 (0.1-7.4)	4.1 (0.3-7.9)	20.4 (11.8-29.0)	24.0 (14.7-33.3)	89.5 (71.5-107.5)	107.8 (88.1-127.5)
2013	4.7 (0.6-8.8)	5.1 (0.8-9.4)	15.7 (8.1-23.3)	18.3 (10.2-26.4)	94.2 (75.7-112.7)	110.7 (90.7-130.7)
Aver age	3.0 (0.3-6.3)	3.5 (0.2-7.0)	22.9 (13.8-32)	30.5 (20.1-40.9)	96.4 (77.7-115.1)	127.4 (106.1-148.7)
β*	0.13	0.08	-1.94	-3.29	-2.27	-5.63
P**	0.162	0.450	0.002	0.001	0.093	0.007

OR: Odds ratio; CI: Confidence interval; SAH: Subarachnoid hemorrhage; ICH: Intracranial hemorrhage; IS: Ischemic stroke; CIR: Crude incidence rate; AIR: Age-adjusted incidence rate; β: Regression coefficient

*Regression coefficient;

**Chi-square test (AIR values are adjusted by the Segi’s world population^12^)

**Table 3:** Crude incidence rate (CIR) (per 100000) and age-adjusted incidence (AIR) (per 100000) of stroke in men, women, and total population in Isfahan, Iran, during 2003-2013

**Year**	**Men [OR (95% CI)]**			**Women [OR (95% CI)]**			**Total [OR (95% CI)]**		
	**n**	**CIR**	**AIR**	**n**	**CIR**	**AIR**	**n**	**CIR**	**AIR**
2003	1253	152.6 (129.9-175.3)	298.0 (268.5-327.6)	1303	167.4 (143.7-191.1)	167.4 (143.7-191.1)	2556	159.8 (135.8-183.8)	229.8 (201.5-258.2)
2004	1190	142.4 (120.5-164.3)	275.6 (247.1-304.2)	1263	159.2 (136.1-182.4)	226.6 (199.9-253.4)	2453	150.6 (127.3-173.9)	213.0 (185.7-240.4)
2005	1225	144.0 (121.9-166.1)	198.9 (173.6-224.2)	1310	162.1 (138.8-185.5)	227.7 (200.8-254.6)	2535	152.8 (129.3-176.3)	212.8 (185.4-240.2)
2006	1127	130.2 (109.2-151.2)	224.6 (198.3-250.8)	1218	148.0 (125.7-170.4)	202.7 (177.2-228.2)	2345	138.9 (116.5-161.3)	190.3 (164.4-216.2)
2007	988	111.8 (92.3-131.3)	183.1 (159.1-207.0)	908	108.4 (89.3-127.6)	145.7 (124.0-167.4)	1896	110.2 (90.2-130.1)	147.3 (124.4-170.1)
2008	920	102.9 (84.2-121.6)	170.8 (147.6-193.9)	904	106.1 (87.2-125.1)	137.4 (116.2-158.5)	1824	104.5 (85.0-123.9)	136.5 (114.5-158.5)
2009	807	86.8 (69.5-104.1)	138.2 (117.2-159.1)	726	77.8 (61.4-94.1)	101.7 (83.4-120.1)	1533	86.4 (68.7-104.1)	111.3 (91.4-131.2)
2010	1231	127.2 (106.3-148.1)	211.0 (185.1-236.9)	1115	110.4 (90.8-129.9)	142.1 (120.2-163.9)	2346	130.3 (108.6-152.1)	165.2 (140.9-189.5)
2011	1127	136.2 (114.8-157.6)	213.9 (188.1-239.7)	1037	116.3 (96.4-136.2)	142.9 (121.1-164.6)	2164	126.5 (105.1-147.8)	157.2 (133.5-180.9)
2012	1201	127.1 (106.2-148.0)	195.1 (170.1-220.2)	1050	116.2 (96.3-136.1)	138.5 (117.0-160.0)	2251	121.8 (100.7-142.8)	145.6 (122.8-168.5)
2013	1195	125.0 (104.3-145.7)	187.3 (162.7-212.0)	1088	118.9 (98.7-139.1)	138.8 (117.2-160.4)	2283	122.0 (101.0- 143.1)	142.9 (120.2-165.6)
Average		126.0 (105.4-146.7)	208.8 (183.4-234.1)	-	126.4 (105.9-147.0)	161.0 (138.3- 183.8)	-	127.6 (106.2-149.0)	168.4 (144.1-192.7)
β*		-2.3	-8.2	-	-5.9	-8.3	-	-3.8	-8.7
P**		0.210	0.050	-	0.021	0.019	-	0.058	< 0.009

OR: Odds ratio; CI: Confidence interval; CIR: Crude incidence rate; AIR: Age-adjusted incidence rate; β: Regression coefficient

* Regression coefficient;

** Chi-square test (AIR values are adjusted by the Segi’s world population^12^)

**Figure 1:**
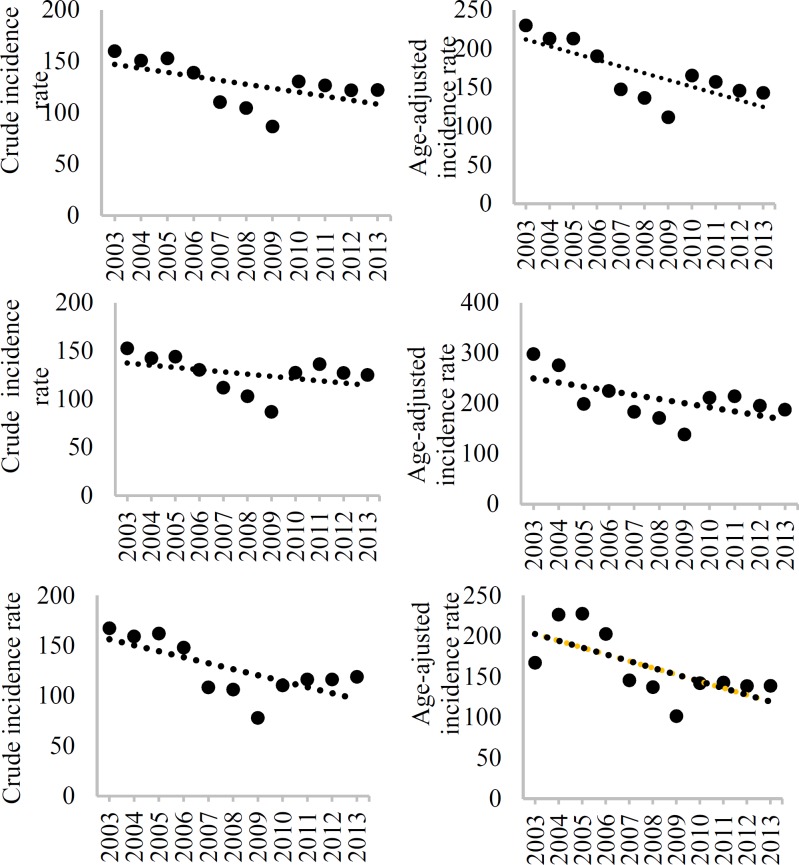
Crude incidence rate (CIR) and age-adjusted incidence rate (AIR) (per 100000) of all stroke types among the total (A), men (B), and women (C) in Isfahan, Iran, from 2003 to 2013

**Figure 2:**
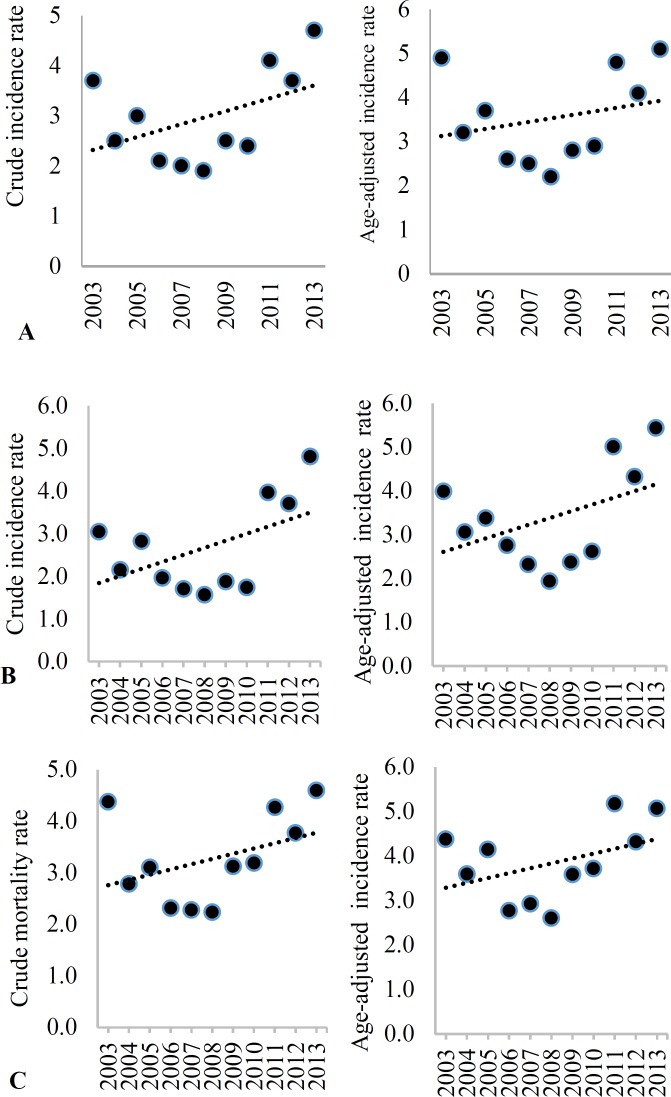
Crude incidence rate (CIR) and age-adjusted incidence (AIR) (per 100000) of subarachnoid hemorrhage (SAH) among the total (A), men (B), and women (C) in Isfahan, Iran, from 2003 to 2013

**Figure 3:**
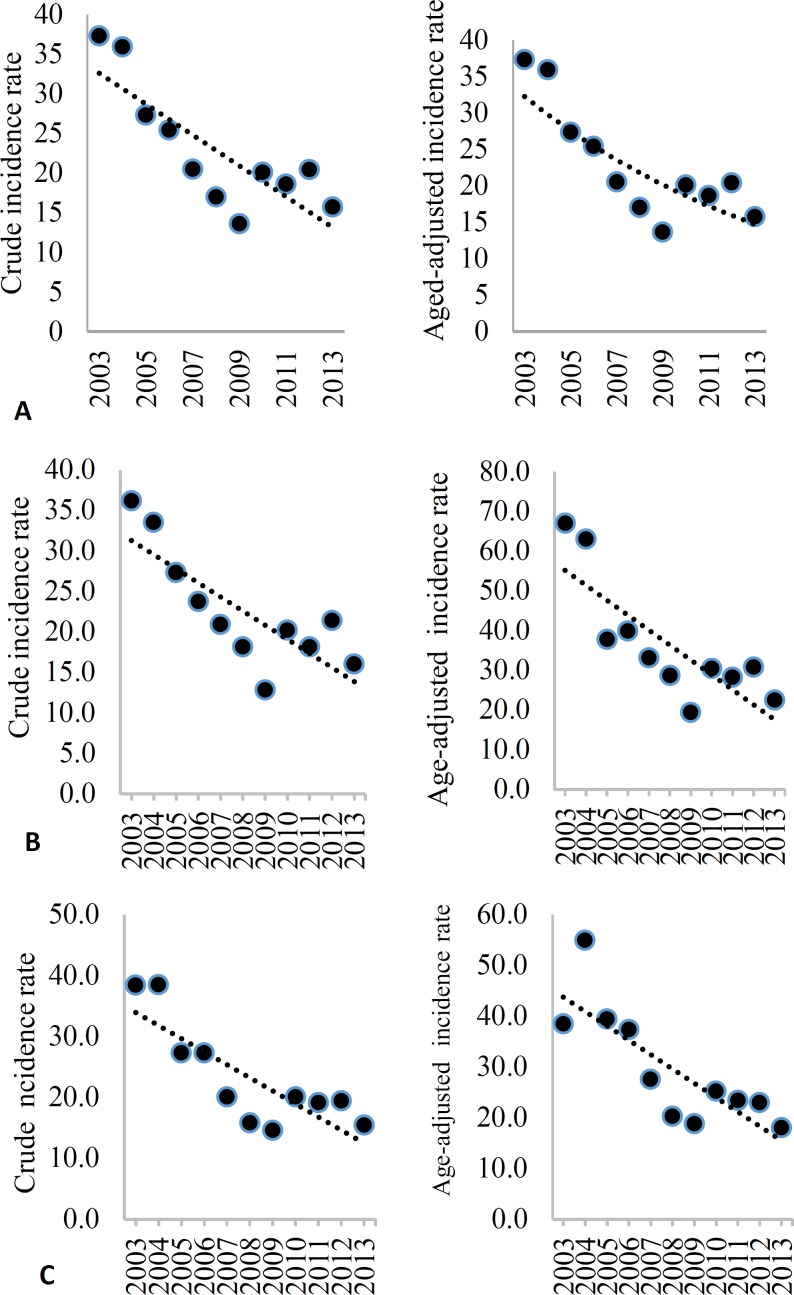
Crude incidence rate (CIR) and age-adjusted incidence (AIR) (per 100000) of intracranial hemorrhage (ICH) among the total (A), men (B), and women (C) in Isfahan, Iran, from 2003 to 2013

**Figure 4:**
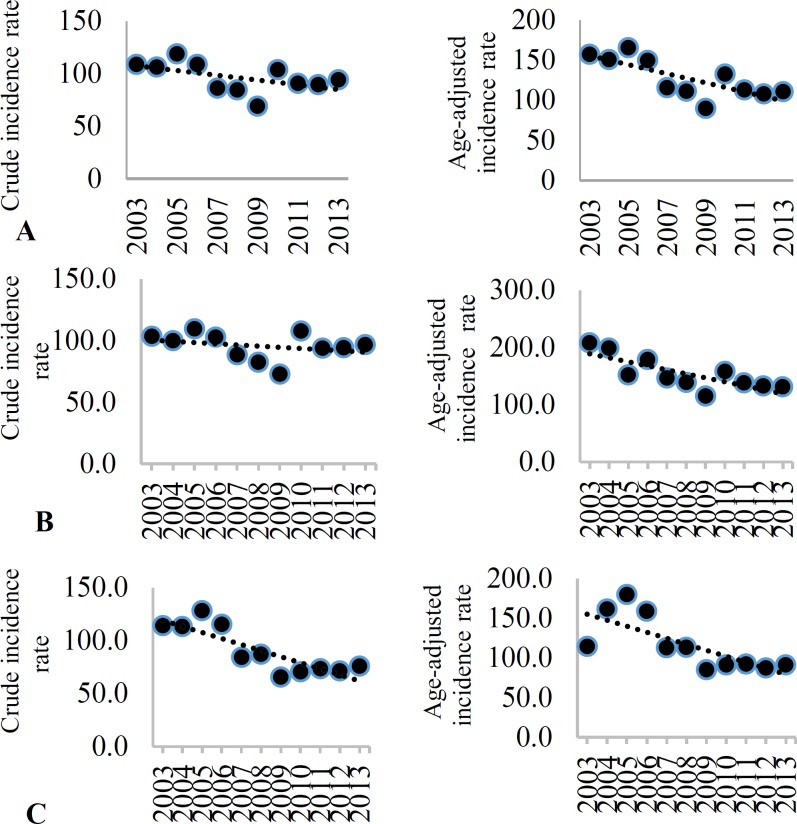
Crude incidence rate (CIR) and age-adjusted incidence (AIR) (per 100000) of ischemic stroke (IS) among the total (A), men (B), and women (C) in Isfahan, Iran, from 2003 to 2013

Table 3 shows stroke CIR and AIR (per 100000 population) during 2003 to 2013. The mean CIR and AIR of stroke in both sexes over the study period were 127.6/100000 (95% CI: 106.2-149.0) and 168.4/100000 (95% CI: 144.1-192.7), respectively. Men had a higher AIR rate of stroke compared to women (208.8/100000, 95% CI: 183.4-234.1 vs. 161.0/100000, 95% CI: 138.3-183.8, respectively), but this was not observed in CIR, with 126.0/100000 (95% CI: 105.4-146.7) for men and 126.4/100000 (95% CI: 105.9-147.0) for women. Overall, the CIR and AIR rate of stroke decreased from 2003 to 2013 (CIR, β = -3.8 and AIR rate, β = -8.7), but this trend was significant only in crude incidence (P = 0.009). Similarly, there was a decreasing trend in the CIR and AIR of stroke in both sexes from 2003 to 2013; however, this trend was significant only for women (Table 3). Figures 1-4 show the trends of CIR and AIR (per 100000 population) of stroke and its subtypes among the total population, men, and women, respectively. As can be seen, decreasing trend was seen in incidence rates during the study period, except for SAH.

**Figure 5:**
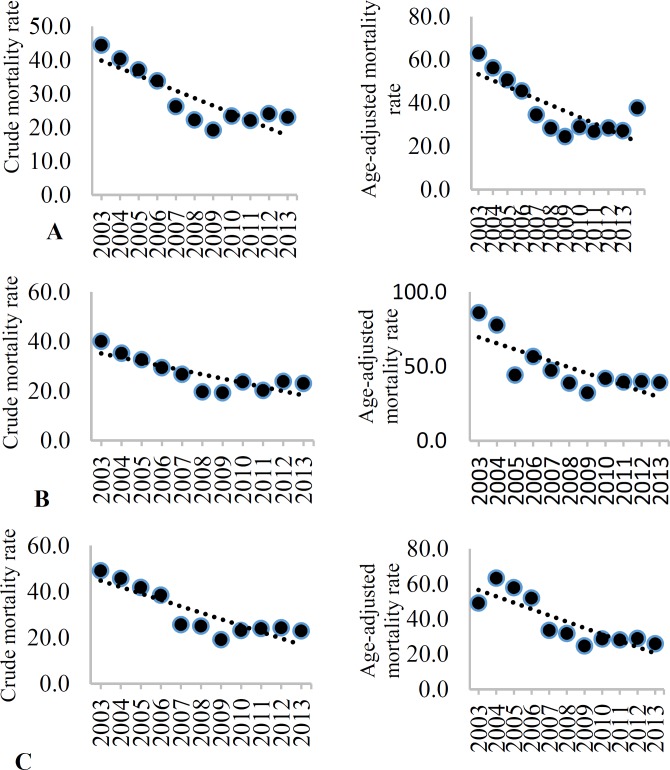
Crude incidence rate (CIR) and age-adjusted incidence (AIR) (per 100000) of all stroke types among the total (A), men (B), and women (C) in Isfahan, Iran, from 2003 to 2013

**Figure 6:**
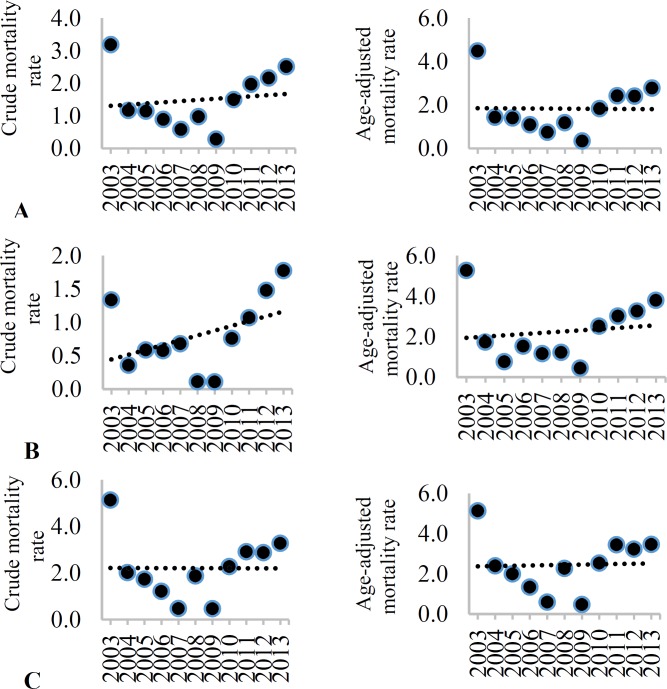
Crude incidence rate (CIR) and age-adjusted incidence (AIR) (per 100000) of subarachnoid hemorrhage (SAH) among the total (A), men (B), and women (C) in Isfahan, Iran, from 2003 to 2013

**Figure 7:**
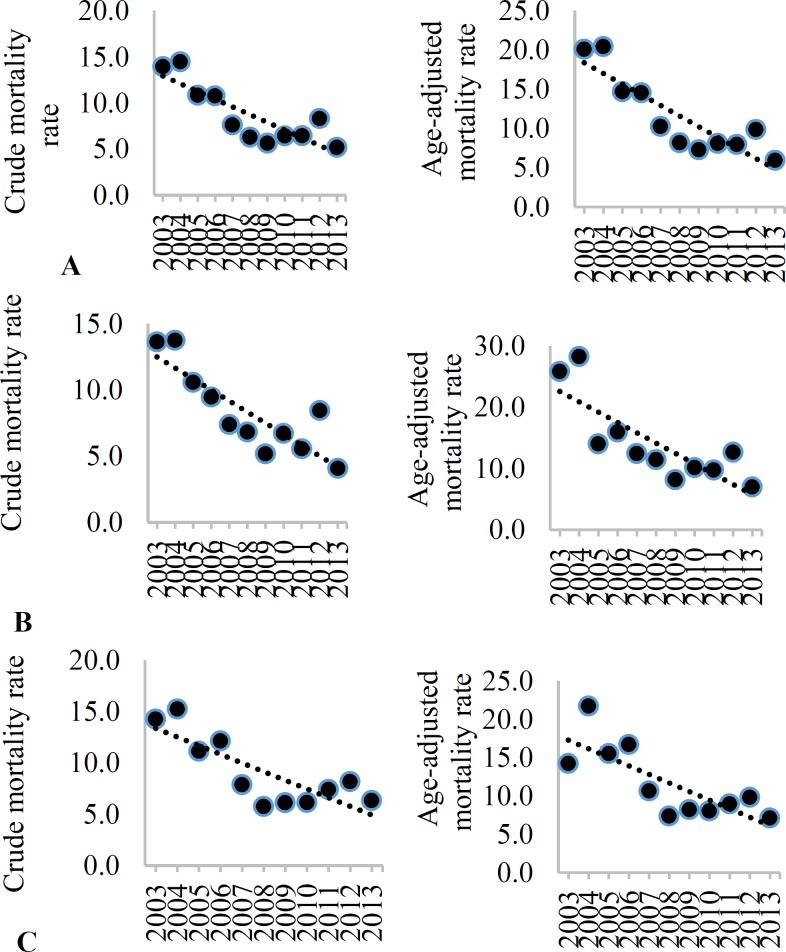
Crude incidence rate (CIR) and age-adjusted incidence (AIR) (per 100000) of intracranial hemorrhage (ICH) among the total (A), men (B), and women (C) in Isfahan, Iran, from 2003 to 2013

**Figure 8:**
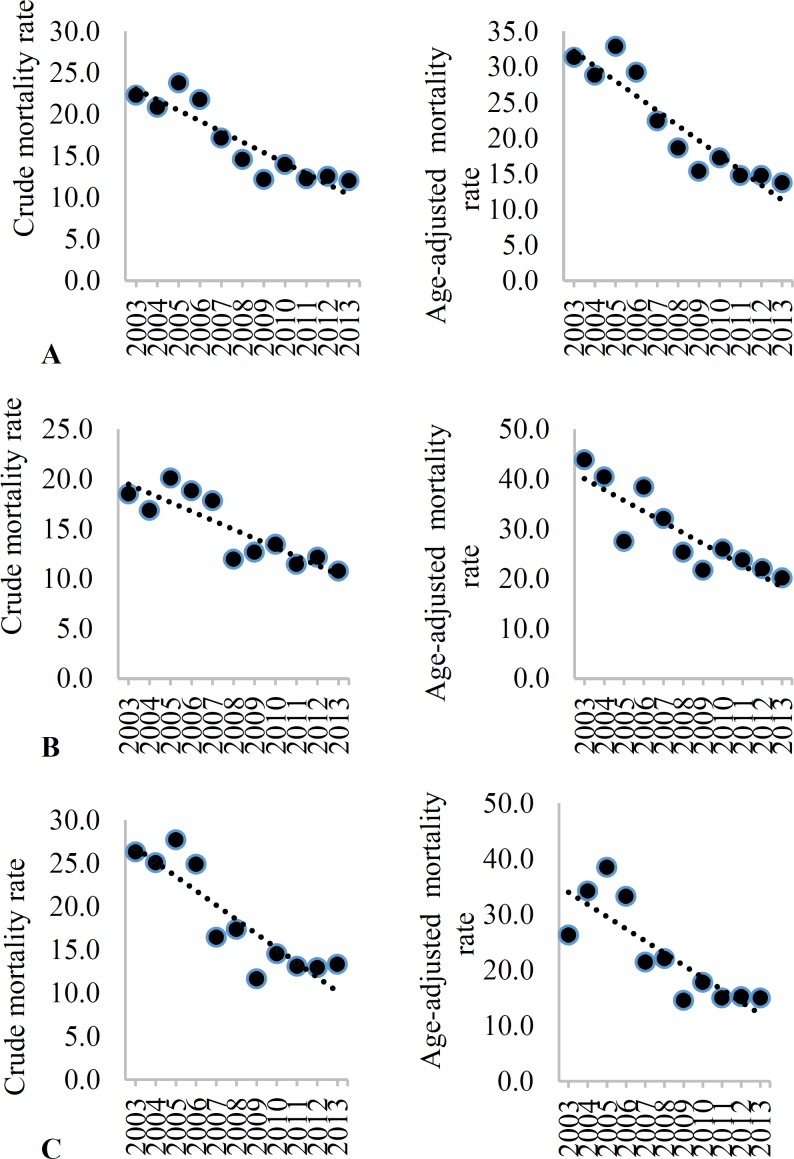
Crude incidence rate (CIR) and age-adjusted incidence (AIR) (per 100000) of ischemic stroke (IS) among the total (A), men (B), and women (C) in Isfahan, Iran, from 2003 to 2013

Figures 5-8 show the trend of crude and age-adjusted mortality rates (per 100000 population) of stroke and its subtypes among the total population, men, and women, respectively. Decreasing trend was visible in mortality rates among the total population, men, and women in Isfahan, during the study period.

As shown in table 4, men were less likely to die due to stroke compared to women (OR = 0.81, 95% CI: 0.75-0.87, P < 0.001). With increased age in patients with stroke, the mortality OR increased (OR = 1.04, 95% CI: 1.03-1.05, P < 0.001). Stroke mortality rate was lower in individuals from urban areas than rural areas (OR = 0.76, 95% CI: 0.69-0.85, P < 0.001). 

The odds ratios (OR) of 28-day mortality due to ICH (OR = 4.04, 95% CI: 3.71-4.41, P < 0.001) and SAH (OR = 3.94, 95% CI 3.20-4.86, P < 0.001) were higher than IS (as reference).

The stroke mortality risk in patients with TIA was higher than the patients without it (OR = 1.31, 95% CI: 1.20-1.41, P < 0.001). The stroke mortality and risk in patients with a history of heart attack were higher than in patients without a history of heart attack (OR = 1.46, 95% CI: 1.35-1.57, P < 0.001 and: OR = 1.29, 95% CI: 1.14-1.45, P < 0.001, respectively).

## Discussion

This was a retrospective, non-community epidemiologic study on stroke and its subtypes in Isfahan. Overall, over the 10-year period of 2003-2013, a decreasing trend in stroke incidence was observed among both sexes. Indeed, AIR decreased from 229.8/100000 in 2003 to 168.4/100000 in 2013. In recent decades, several studies have reported a decline in stroke incidence rate^14^^-^^16^ which agrees with our results.

On the other hand, this finding is not consistent with previous studies in some cities of Iran that have reported an increasing trend of stroke in recent years.^8^^,^^17^

It is worth noting that the duration of previous studies conducted in this regard in Iran was between one to six years. 

**Table 4:** Logistic regression analysis for the association of sex, age, living area and stroke risk factors for stroke incidence and mortality

**Variable**		**28-day mortality**		
		**OR**	**95% Cl**	**P** *
Sex	Woman	1	-	
	Man	0.81	0.75-0.87	< 0.001
Age		1.04	1.04-1.05	< 0.001
Living area	Rural	1	-	
	Urban	0.76	0.69-0.85	< 0.001
Stroke subtypes	IS	1		
	SAH	3.94	3.20-4.86	< 0.001
	ICH	4.04	3.71-4.41	< 0.001
TIA	Yes	1.31	1.20-1.42	< 0.001
	No	1	-	
Diabetes	Yes	1.02	0.94-1.11	0.590
	No	1	-	
High blood pressure	Yes	0.92	0.85-0.99	0.040
	No	1	-	
Hearth attack	Yes	1.46	1.35-1.57	< 0.001
	No	1	-	

* Logistic regression; OR: Odds ratio; CI: Confidence interval; IS: Ischemic stroke; SAH: Subarachnoid hemorrhage; ICH: Intracranial hemorrhage; TIA: Transient ischemic attack

Nevertheless, the mean age-adjusted stroke incidence observed between 2003-2013 in Isfahan (168.4/100000) was high in comparison with previous studies in Iran and some developing countries.^8^^,^^11^^,^^18^ The reason for this large difference with most other countries in the world and region is not clear.

In this study, the crude stroke incidence in each sex, separately and in combination, increased after adjusting for age. The low CIR of stroke indicated that our study population is young.^8^ The CIR of stroke in women (126.4/100000) was slightly higher than men (126.0/100000), whereas this was reversed after adjusting for age. This indicates a higher incidence of stroke among older women than men. As was observed in this study, overall, women were older than men, which is consistent with the findings of Di Carlo, et al.^19^ in a study involving 7 European countries.

In this study, among all types of stroke, IS was predominant stroke subtype, which was in line with previous studies from Iran.^8^^,^^17^ In addition, high blood pressure had the highest frequency among other stroke risk factors which is in line with previous studies.^20^^,^^21^

Despite the decreasing trend of IS and ICH incidence during 2003-2013 in Isfahan, there was a significant increasing trend in SAH subtype incidence. It seems that SAH subtype is more common in younger populations^11^^,^^22^ that may explain the increasing trend in this study.

In line with the other studies, results of this study showed the higher mortality risk among women.^23^

The reason for high mortality risk could be explained by the higher mean age of female patients as well as higher proportion of all risk factors among them. Furthermore, some studies pointed out that women have worse recovery than men after stroke.^23^^,^^24^

We found higher stroke mortality risk in rural areas compared to urban areas. Individuals from urban areas are more likely to have access to healthcare services before the stroke, such as continuous monitoring of blood pressure, than those from rural areas. On the other hand, after the occurrence of stroke, urban residents are more likely to receive stroke care than rural residents.^5^

In the current study, patients with ICH had a higher risk of stroke mortality followed by SAH and then, IS. These results were consistent with previous studies that identified ICH as deadliest form of stroke.^25^^,^^26^

Overall, the risk factors examined in this study showed a significant effect on stroke mortality and incidence risk. Among the risk factors, blood pressure with greater proportion than other risk factors appears to play an important role in stroke incidence.^27^ Nevertheless, the result obtained in the present study, showing high blood pressure reduced the risk of stroke mortality, was somewhat unexpected and indicated a need for further investigation.

Based on our findings, diabetes mellitus, and history of a heart attack can be suggested predictors of risk of stroke and mortality.

## Conclusion

The present study provided a comparative and longitudinal study of stroke incidence in Isfahan. Overall, despite the relatively high incidence of stroke over the study period, the stroke incidence rate had a decreasing trend over the past decades in Isfahan, especially in ICH subtype. However, given the young population of Iran, there will be a larger elderly population than expected in the coming years. On the other hand, due to the lack of appropriate control of risk factors in our elderly population, it could be expected that the stroke incidence would be rising in the future. Because of the high frequency of stroke, policy should focus on developing stroke care interventions as well as raising awareness about the risk factors.

## Notes:

**How to cite this article:** Bahonar A, Khosravi A, Khorvash F, Maracy M, Oveisgharan S, Mohammadifard N, Saadatnia M, Nouri F, Sarrafzadegan N. Ten-year trend in stroke incidence and its subtypes in Isfahan, Iran during 2003-2013. Iran J Neurol 2017; 16(4): 201-9.
